# Digital transformation, well-being and shrinking communities: Narrowing the divides between urban and rural

**DOI:** 10.1016/j.heliyon.2023.e18801

**Published:** 2023-08-06

**Authors:** Annamari Kiviaho, Johannes Einolander

**Affiliations:** a Ruralia Institute, University of Helsinki, Lönnrotinkatu 7, 50100, Mikkeli, Finland; b Department of Built Environment, Aalto University, School of Engineering, P.O. BOX 15800, FI-00076, Aalto, Finland; c Department of Mechanical Engineering, Aalto University, School of Engineering, P.O. BOX 14100, FI-00076, Aalto, Finland

**Keywords:** Digitalisation, ICT, Wellbeing, Shrinking communities, Remote services

## Abstract

The ongoing digital transformation and the digitalisation of services profoundly affect the everyday lives of citizens. Digital transformation's impact is complex and may have both positive and negative consequences on well-being. Particularly in rural areas and shrinking communities, digitalisation and the concentration of services can diminish the level of local services, but digital transformation also promises significant opportunities and benefits to the residents of shrinking and rural communities. This study employed the environmental scanning and futures wheel methodologies to assess the current and future impact of digital transformation and information and communications technology on residents' well-being in shrinking Finnish communities. The results indicate that this impact is more positive than negative, as digitalisation enables multiple services that were previously lacking in these locations. Digitalisation and the rise of remote working can also bring new opportunities and residents to rural and shrinking communities.

## 1 Introduction

Digitalisation and the diverse implementation of information and communications technology (ICT) have accelerated in recent years, with the Covid-19 pandemic hastening the process even further, and this digital transformation profoundly affects people's everyday lives. Previous studies on digitalisation's impacts and consequences for citizens are related mostly to growing cities and especially so-called smart cities but the residents of rural and shrinking communities are also greatly affected by digitalisation.

The term *shrinking cities* describes cities and areas suffering from significant population decline [1]. In many academic papers, the term embraces several macro-trends and causal factors in urban development, such as economic downturns, growing unemployment, outmigration and social problems [1]. Shrinkage is a common phenomenon and has long been a significant challenge in many countries around the world [2]. For instance, Wolff and Wiechmann observe that one-fifth of European cities experienced population loss between 1990 and 2010 [3]. Cities and regions are also shrinking in rapidly urbanising developing countries [4]. Population loss does not affect only cities, however, and rural municipalities, for instance, may also experience shrinkage.

Previous studies indicate that population decline, including urban shrinkage [1], results from a combination of different factors. Some researchers contend that economic decline due to deindustrialisation causes population loss in shrinking regions, resulting in job losses and unemployment and thus intensifying job seekers' out-migration [5,6]. Natural demographic changes, such as an ageing population and low fertility rates, are among the factors driving population loss in shrinking regions [6]. ‘Shock’ events, such as natural disasters and wars, also cause population reduction as noted by Wiechmann and Bontje [7]. Population decline has multiple negative consequences, such as high vacancy rates [8], abandoned properties [1] and underused utilities and transportation networks [9]. Shrinkage also has many negative socioeconomic impacts, including increased poverty and segregation [10], unemployment, job losses and reduced tax bases and local services [11]. Many of these negative consequences endanger the welfare of the community and the well-being of residents.

The umbrella terms *well-being* and *quality of life* cover multiple concepts [12] and embrace similar elements and meanings that may partly overlap, causing terminological confusion. Two conceptual approaches address well-being: subjective and objective well-being [13]. The objective approach measures well-being by various indicators that can be seen as components of a good life, such as income, food and housing [13]. Subjective well-being encompasses people's subjective evaluations and experiences of diverse aspects of their lives [14].

The relationship between subjective well-being and various factors has been studied for many years. For instance, Blanchflower and Ostwald identify a positive relationship between subjective well-being and level of education [15]. Other factors that improve subjective well-being include membership in organisations [16], physical and psychological health [17] and higher income. Researchers have also identified factors with a negative impact on well-being, such as unemployment [18] and long commutes [19].

Several previous studies have shown that digitalisation and ICT can have many positive impacts on well-being. For instance, Maiti and Awasthi employed an index of ICT exposure to study its effects on well-being and found that ICT exposure improves well-being at the aggregate level [20]. The authors of [21] studied the connection between ICT and well-being through a literature review, finding that ICT influences people's well-being in many ways, such as by saving time, facilitating access to information, creating new activities and improving communication. Many studies have shown that digitalisation and the implementation of various ICT technologies positively influence the well-being of the elderly [[22], [23], [24]]. However, digitalisation can also have negative effects on elders' well-being. For instance, Vilpponen et al. claim that digitalisation increases anxiety in the elderly and thus undermines their well-being [25]. Other studies have shown that the use of ICT technologies can adversely affect well-being. For example, research has linked the use of ICT in organisations to stress, technostress, anxiety, strain and burnout [26,27]. According to Pfaffinger et al. using ICT can blur the line between the home and work environments, causing employees to feel constantly connected to their work [28] and making it challenging for them to mentally disconnect and recuperate from work. Similarly, the use of social media has been associated with negative impacts on well-being [29,30].

This study focused on the impact of digital transformation and digitalisation on the well-being of the residents of shrinking communities. The well-being of residents of shrinking cities has been previously studied (e.g. Refs. [31,32]), but the impact of digitalisation and ICT has not been comprehensively examined in the context of shrinking communities. Studies are basically non-existent on smaller, rural shrinking communities and digitalisation's impact on their residents' well-being. However, digitalisation has enabled small and rural communities to use services previously out of reach for their residents, and more extensive research is needed to assess the possible consequences.

This paper aims to advance the understanding of digitalisation's impact on the well-being of shrinking communities now and in the future, thus bridging the existing research gap. The study employed futures research methods and took Finland as a case study to assesses digitalisation's current and future consequences on well-being, using environmental scanning and content analysis to examine 600 local and national newspapers to form a comprehensive understanding of the current state of affairs. Conceptual analysis and futures wheels were also used to support more thorough analysis and visualisation. Overall, the results suggest that digitalisation's impact on the well-being of shrinking and rural communities can be even greater than for residents of large and growing cities.

## 2 Methodology

This study used methods drawn from the field of futures research to assess the impact of digital transformation and ICT on residents’ well-being in shrinking communities. *Futures research* may be defined as critical, systematic, holistic and multidisciplinary research on topics related to the future. In futures research, knowledge of the present is used as evidence to make statements about the future that aim to reveal directions for future development [33]. As futures research is based on observations of the past and present, it provides important information about the current situation in addition to information about possible changes in the future [34]. Therefore, it represents an important instrument for a wide range of planning and decision-making processes [35]. In addition, futures research provides valuable information on future threats and opportunities [34]. Having information about plausible future threats and opportunities makes it possible to better prepare for them. The potential impact of future threats can be mitigated, and impending opportunities can be harnessed to support desired or preferable future development [34].

This study used the environmental scanning method in data collection and employed conceptual analysis and the futures wheel method in data analysis and the assessment of digitalisation's future impacts on the well-being of shrinking communities. These methods and this study's case country are introduced in the following subsections.

### 2.1 Environmental scanning

Environmental scanning involves extensive information gathering and the analysis of specific events or topics and their relationships. It aims to uncover and analyse the forces of change affecting the studied topic, e.g. trends, megatrends, wild cards, weak signals and driving forces [36]. Environmental scanning is usually conducted with data gathered from either textual, human or online sources [37]. In the scanning process, various phenomena are identified and collected, and the development of these phenomena and interactions between them are followed and analysed [36]. By paying attention to the consequences of diverse decisions and events, environmental scanning can explain the changes in various phenomena in a specific environment [37]. Environmental scanning adopts a holistic research perspective, with diverse future consequences being examined comprehensively and on a broad scale [38].

According to Naisbitt, a well-known researcher in the field of futures studies, environmental scanning can provide an awareness of future changes, and the ability to anticipate the consequences of those changes gives actors the opportunity to steer their own actions in a more desirable direction [39]. For this reason, environmental scanning is widely used and adapted in the business sector and organisational studies. Through it, organisations can better understand the external changes that affect their operations and react to them in a way that secures or improves their position in the future [40]. The use of environmental scanning in companies and organisations has been examined in multiple studies [[41], [42], [43]]. The method has also been widely used in academic studies, for instance, to reveal the forces of change affecting the future commercial real estate market [44], to study future themes of the cadastral system [45], to envision the future of sustainable tourism [46], to reveal the forces affecting event planning [47] and to analyse the forces of change affecting the future transportation system [48]. Overall, environmental scanning is a useful tool for analysing the forces of change affecting a phenomenon of interest. In this study, it was used to identify the forces of change affecting shrinking communities and to analyse themes related to ICT, digitalisation and well-being.

### 2.2 The futures wheel

**Fig. 1:**
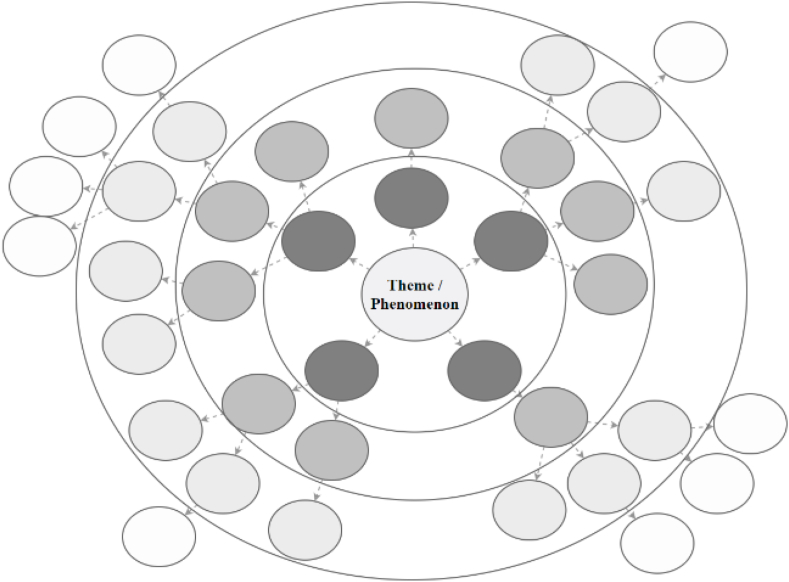
Illustration of the futures wheel methodology.

The futures wheel offers a way to visualise the possible future consequences of a studied phenomenon. The basic method is to place the studied phenomenon at the centre of the wheel and then identify and analyse possible future impacts and consequences of the phenomenon [49]. After the primary impacts of the phenomenon are recorded on the wheel, the idea is to forget the initial phenomenon at the centre of the wheel and focus in a similar fashion on the consequences of every primary impact [49]. These secondary impacts are also recorded on the future wheel, and the process continues similarly to the tertiary level impacts. Finally, after the primary, secondary and tertiary impacts are placed on the wheel, a complex map emerges that describes the implications of the initial phenomenon at the centre. The process can be continued in similar manner until no more consequences or impacts are found. The futures wheel differs from the traditional mind map because it separately identifies the primary, secondary and tertiary (and sometimes further) impacts of the phenomenon [46]. Fig. 1 illustrates how the futures wheel takes shape around the main theme/phenomenon.

One advantage of the futures wheel method is that it reveals the direct and indirect consequences of various types of change [46]. Another advantage is that it allows a transition from linear, hierarchical and simplistic thinking to more network oriented and complex thinking [49]. As a result, unforeseen implications can be found [50].

The futures wheel method has previously been used by a vast range of actors, including military, corporate and public-sector planners and decision-makers [49,50]. It has also been used in multiple academic studies, for instance, to examine the implications of current trends affecting US forests (such as the lack of age-class diversity among trees and their uniform ageing) [51], to discover future trends affecting tourism [46], to reveal future challenges to the mining industry [52] and to study the impacts of the Covid-19 pandemic [53]. In addition, the method has been used in ICT-related studies, including one that analysed the future impacts of ICT tools in education [54].

The futures wheel method can be used in workshops as a means of structural brainstorming and, as noted by Bengston, can be applied to a variety of needs and situations [50]. In some studies, for instance, the wheels were formed by the internal research team based on the data of separate interviews [45,55]. Similarly, in the present study, the futures wheels were formed by the internal research team based on data acquired from environmental scanning.

### 2.3 The case of Finland

A case approach was chosen to demonstrate the impacts of digitalisation on the well-being of shrinking communities, as it produces an in-depth understanding of a phenomenon in its real-life context [56]. The effective and widescale use of remote services and other digitalisation-enabled processes is highly dependent on reliable, high-speed internet connections, however, so, to assess the current and future impacts of digitalisation in shrinking and rural areas, the case locations had to have extensive mobile broadband coverage.

The case example of this study covers eight shrinking municipalities in Finland, a highly digitally advanced but sparsely populated country with many municipalities experiencing population decline. The populations of the country's shrinking communities are also rapidly ageing, as the young tend to move to larger cities in search of work and educational opportunities. It has been estimated that, between 2010 and 2020, almost 75% of Finnish municipalities were losing population [56], and the number of shrinking municipalities is expected to increase in the future. It is also believed that the populations of 80% of Finnish municipalities will decline between 2021 and 2040 [57]. Overall, Finland's cities and municipalities are generally small and sparsely populated [58].

**Fig. 2:**
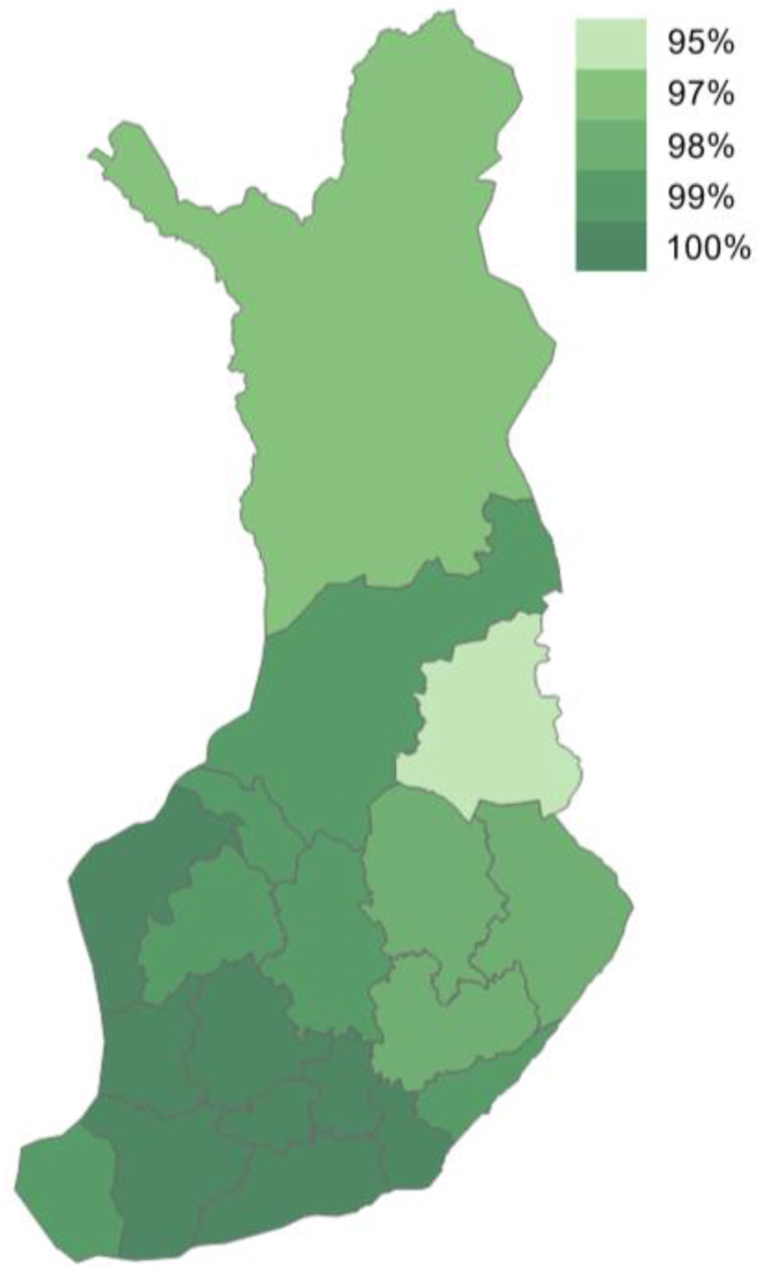
4G mobile broadband (30 Mbps) coverage of Finnish households by region.

Finland already has the infrastructure to support the widescale adoption of digitalisation-related ICT technologies. For instance, 4G mobile broadband at 30 megabits per second (Mbps) reaches 99% of Finnish households, while 94% of households enjoy 4G service at 100 Mbps [59], and high-speed 5G mobile networks covered 84% of Finnish households in the first quarter of 2022 [60]. Fig. 2 illustrates the 4G mobile broadband coverage of Finnish households, which is slightly lower in sparsely populated regions.

In Finland, a municipality denotes an administrative unit that geographically includes population centres as well as surrounding rural areas and local centres in those areas. This study focused on small, sparsely populated towns and regions in Finland because such municipalities suffer the most from population shrinkage. The eight case municipalities were selected on the basis of three criteria: (a) the population had declined more than 10% since 1990, (b) shrinkage had been continuous over 20 years and (c) the municipality had a local newspaper. The selected municipalities are shown in Table 1.

**Table 1:** Case municipalities.

**Municipality**	**Local newspaper**	**2021 population**	**Population change (%) 1990**–**2021**	**Continuous shrinkage period 1990–2021 (years)**	Population density* 2021
Imatra	Uutisvuoksi	25.655	−23.5	1990–2021 (31)	165.5
Jämsä	Jämsän seutu	19.767	−24.0	1994–2021 (27)	12.6
Kemi	Lounais-Lappi	19.982	−21.3	1990–2021 (31)	209.6
Kouvola	Kouvolan sanomat	80.454	−14.7	1993–2021 (28)	31.5
Kurikka	Kurikka-lehti	20.197	−24.0	1990–2021 (31)	11.7
Lieksa	Lieksan lehti	10.543	−39.8	1990–2021 (31)	3.1
Pieksämäki	Pieksämäen lehti	17.253	−28.5	1990–2021 (31)	11.0
Savonlinna	Savonmaa	32.547	−21.3	1996–2021 (25)	14.5

*Residents/square kilometer.

### 2.4 Implementation of the methods

The textual sources reviewed during the environmental scanning included all the local newspapers in the case municipalities and the largest national newspapers in Finland. The national journals comprised the seven largest national newspapers by readership and two electronic news sites published by Finland's largest news media groups. These newspapers were selected to obtain a broader perspective reflecting views and attitudes related to shrinking cities, specifically from a national perspective. Because the case municipalities are small, many support only one local newspaper reporting the municipality's news, so all the local newspapers in the case municipalities were reviewed. Local newspapers were chosen as research material because they can be considered the most important forums for public discourse in the case areas. The scanning was conducted in 2020. Search terms related to urban shrinkage, such as *population decline*, *declining city*, *shrinking city*, *shrinking municipality* and *population loss*, were used to find articles in the national newspapers, whereas the local newspapers were systematically examined with a focus on articles related to changes, events and phenomena in the operating environment. The local newspapers were published between January 2019 and September 2020 and the national newspapers between January 2011 and September 2020. Overall, 642 newspapers were scanned.

All the discovered articles were read, and key themes, topics and issues related to the research environment were identified. During the scanning, more than 2000 observations were made and recorded to a database for further analysis. Table 2 demonstrates the structure of the database. The observations included the forces of change affecting shrinking communities, such as trends, megatrends, wild cards, driving forces and weak signals. The recorded observations describe changes, events and phenomena and how they manifested in the research environment. As in another study [45], the observations could be a news item, a topic covered in the news or a phrase used in the news. The observations were examined by content analysis and categorised based on their content. Next, the non-ICT related observations were discarded, and the ICT-related observations (16% of all observations) were reanalysed, coded and categorised into primary categories and broader themes.

**Table 2:** Example of the database.

**Observation**	**Title of the article**	**Data source**	**National/Local newspaper**	**Publishing date of the newspaper**
A remote doctor in response to the shortage of doctors	“In response to the shortage of doctors, a remote doctor was brought in to help - they will provide care two days a week."	Kurikka-lehti	Local	17/10/2019
“Online stores and brick-and-mortar stores live in symbiosis, as products are bought online but are returned or exchanged at the physical store."	“Loyal customers brought the store back to Pieksämäki, brick-and-mortar stores also support online sales"	Pieksämäen lehti	Local	05/05/2019
“The respondents of the survey felt that the opportunity to work remotely and the flexibility of working life increase well-being both in the workplace and outside of it."	“Employees want to work remotely - There are geographical differences in the opportunities."	Kauppalehti	National	16/12/2019
Multi-local living is becoming more common in rural areas that are facing challenges of popution decline	“Rural areas and cities progress together in sustainable development - they are not in competition with each other."	Maaseudun tulevaisuus	National	25/02/2017
…	…	…	…	…

Four main themes relating to the impact of digitalisation on the well-being of shrinking and rural communities—remote working, distance learning, distance services and e-commerce—emerged from the conceptual analysis of the data and were subjected to closer relational analysis. Futures wheels were employed as a visualisation and analysis tool to address the current and future impacts of digitalisation on shrinking communities’ well-being. The futures wheel was chosen as the method as in many similar studies (e.g. Refs. [45,55]) it has been used as an appropriate method for data analysis and visualisation, as it offers a structured, organised way to explore complex relationships between relational phenomena. In this study, a team of experts analysed the data by main themes and examined the relationships between subthemes and concepts in the processed articles, leading to the creation of the futures wheels. That is, environmental scanning was used to generate the initial database, and the expert team employed content, conceptual and relational analysis to categorise and assessed the primary, secondary and tertiary impacts of each main theme.

## 3 Results

This section provides an overview of the study's findings, focusing on the four main themes that were selected for closer analysis. We first describe the consequences associated with remote working, followed by the potential future impacts of distance learning and remote services. Lastly, we describe the impacts of e-commerce.

### 3.1 Remote working

A main cause of shrinkage in many cities and communities is a lack of job opportunities. Most shrinking communities have high unemployment rates, so the population decline is accelerated by the out-migration of job seekers, but this study's results suggest that the ongoing rise of remote working may counter these trends and bring new job opportunities to residents of shrinking communities.

**Fig. 3:**
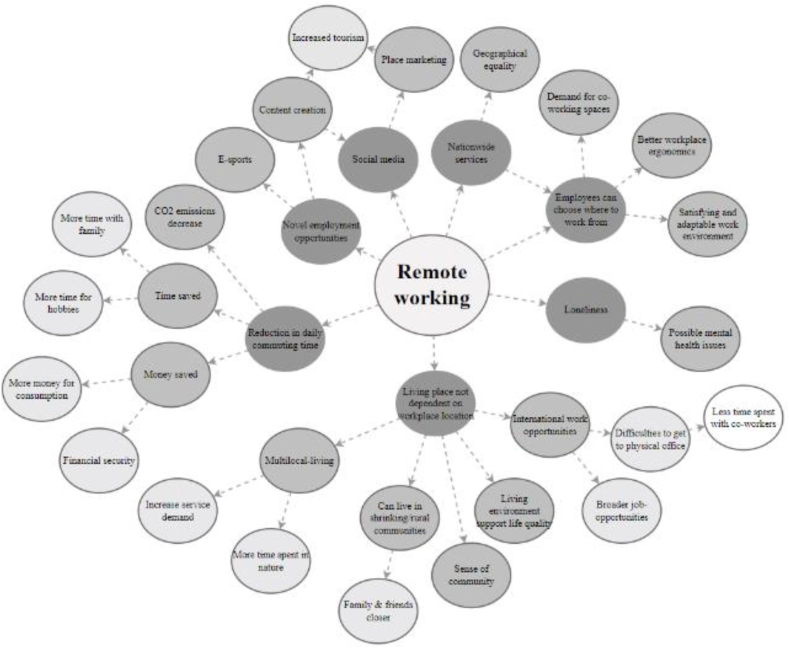
Simplified futures wheel for remote working.

Fig. 3 presents a futures wheel showing the network of consequences that remote working may have on the well-being of residents of shrinking communities. The primary impacts of remote working are visualised with a dark colour and other consequences with lighter colours. Due to the complicated and wordy nature of the various consequences and the difficulty of describing them succinctly, the futures wheel is presented in a somewhat simplified form. The most frequent concepts are discussed in the following paragraphs.

Remote working enables multi-local living and makes it possible for employees to live for longer periods in locations farther from their workplace, for example, in a second home or summer cottage in the countryside. This is especially true in Finland, which has a long tradition of recreational second homes and summer cottages. Remote working makes it possible to work while staying at these second homes, which are generally located in the heart of nature, a condition generally observed to improve overall well-being in humans. An increase in multi-local living also benefits shrinking municipalities with large concentrations of second homes, as the second homeowners maintain the demand for services and enliven the communities.

Remote working also reduces the need for daily commuting, saving people both time and money. In particular, the time saved from commuting can be used for more meaningful activities supporting overall well-being, such as hobbies, time with the family and other activities. The money saved from commuting can increase financial security or be directed to consumption that improves overall well-being.

Although remote working reduces daily commuting, the length of commutes might increase as location-independent remote working enables people to live farther away and even in different countries than the physical location of the workplace. At the same time, remote working allows people to choose the location of their home or place of residence regardless of the location of the workplace, enabling them to choose their place of residence and residential environment according to their preferences. Thus, they can live in environments that match their values and support their quality of life. This can also improve employees’ financial security, as it can be very much cheaper to reside farther from major cities.

The growth of remote working is also changing the needs of home workspaces. In the home office, the employee can determine the space solutions of the workspace, for example, the interior design, ergonomics and overall comfort, which can promote well-being at work. However, some people may suffer from loneliness if they work remotely for long periods.

The Covid-19 pandemic caused a major shift from office work to remote working in Finland. During that time, a new phenomenon emerged of people moving away from major cities to larger homes closer to nature. This caused a net migration gain in some cities and municipalities that were previously shrinking.

Digitalisation has also enabled novel employment opportunities for residents of rural and shrinking communities. For instance, social media content creation and the gaming industry have made self-employment possible for rural youth who otherwise would likely move to larger cities. Content creation by residents of shrinking and rural communities can also attract tourism and new residents and thus support the whole community.

### 3.2 Distance learning

**Fig. 4:**
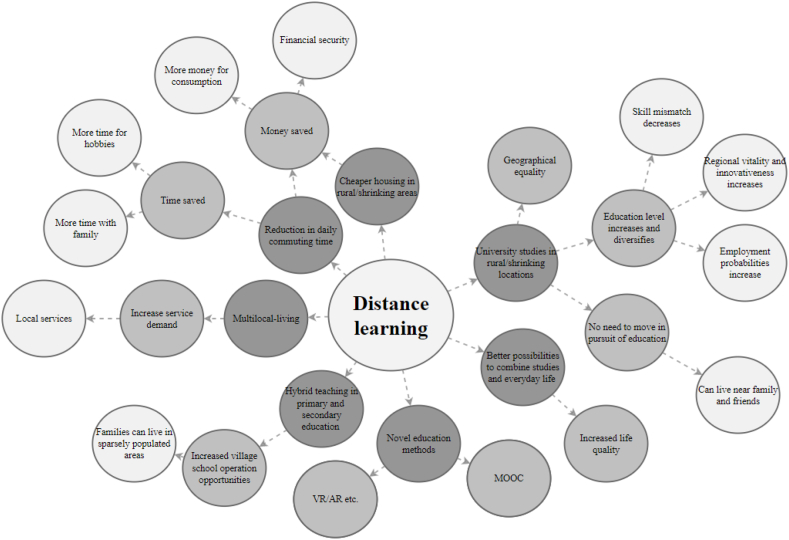
Simplified futures wheel for distance learning.

The education sector has also been affected by digitalisation and ICT. In recent years, many Finnish universities and universities of applied sciences have introduced multiple courses and degrees that can be completed online, enabling flexible, location-independent distance learning. It is also possible to complete the entire upper secondary school course load online in Finland. The simplified futures wheel for distance learning's impacts on the well-being of residents of shrinking communities is presented in Fig. 4. The most prominent themes are analysed below.

Distance learning creates opportunities, especially in shrinking and rural communities, as people do not have to move to another city to study, allowing them to study and even complete university degrees while staying in their hometown close to family, friends and relatives. Distance learning also makes it easier to combine work, studies and family life, which has an impact on overall well-being.

Broadly, easier access to study opportunities through distance learning can increase and diversify the education level of residents of shrinking cities, so employment opportunities and income levels may increase, which can affect overall well-being. A higher education level can also affect the regional development of shrinking cities through increased innovation and a more skilled workforce. Additionally, distance learning reduces the need for daily commuting between home and the university campus, enabling multi-local living as with remote working.

During the Covid-19 pandemic, most Finnish universities and some upper secondary schools took their courses totally online, which accelerated distance learning and improved online education practices. The number of massive open online courses (MOOCs) also grew during the pandemic, making it possible for non-students to study various subjects for free in their homes. Overall, as with remote working, distance learning advances geographical equality and provides previously unavailable opportunities to the residents of shrinking and rural areas.

### 3.3 Remote services

**Fig. 5:**
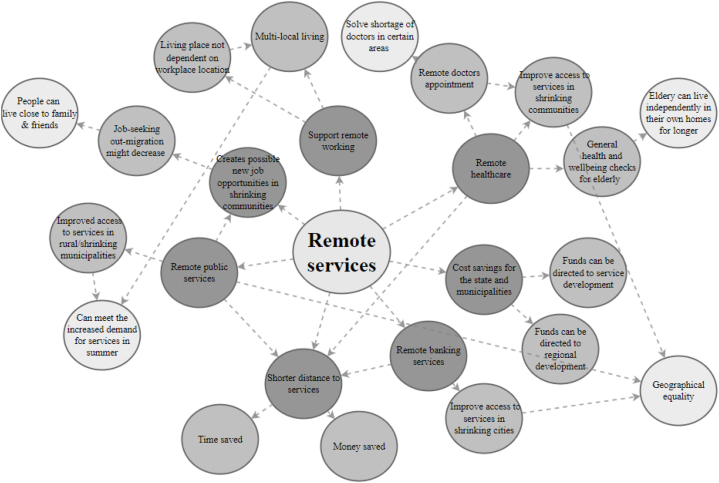
Simplified futures wheel for remote services.

Various remote services recurred in our data, such as remote healthcare, banking and public services. Remote provision improves access to services and thus promotes equality and social justice, especially in rural and shrinking communities, where many services have been cut back due to declining populations and the concentration of state and municipal services. Nowadays, most Finnish public services can easily be conducted remotely online, including municipality services, social security services, public employment and business services, tax administration services and public legal aid services. Fig. 5 presents the futures wheel depicting the impacts of remote services on the well-being of residents of shrinking cities. The most common and impactful themes are discussed in the following paragraphs.

Some shrinking communities have faced significant difficulties in attracting a skilled workforce to the social and healthcare sectors. Many small municipalities, for instance, have struggled to recruit medical doctors. Remote healthcare services can alleviate these challenges, however, as many mild illnesses can be treated remotely, and remote veterinary services can treat minor illnesses and injuries of household pets.

Remote healthcare services especially promote the well-being of the elderly. Using remote connections and virtual visits, healthcare professionals can easily conduct general health and well-being checks on the elderly population far from large city centres, especially in sparsely populated areas. All the aforementioned remote services enable the elderly to live independently for a longer time in their own homes. Not all individuals have the necessary skills to use remote services, which increases societal inequality, but some support and educational schemes are offered for the elderly and others who currently lack the necessary digital skills.

Remote services also support remote working and multi-local living. For example, because many government services can be provided remotely, many government employees can choose their place of residence regardless of the physical location of the workplace. In addition, remote services benefit shrinking cities and municipalities with large concentrations of second homes. The population grows especially significantly in these municipalities in the summer months, and remote services can meet the increased demand.

### 3.4 E-commerce

**Fig. 6:**
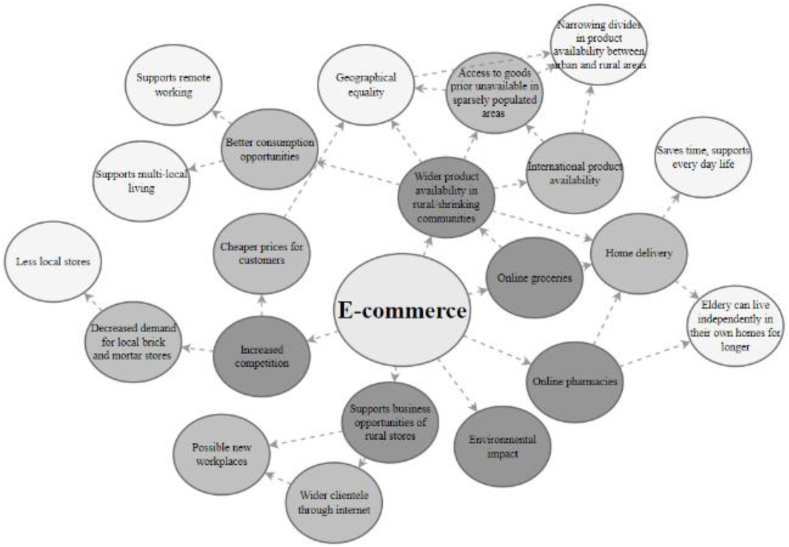
Simplified futures wheel for e-commerce.

E-commerce has grown at a steady pace in recent years, a development accelerated by Covid-19. In shrinking Finnish cities, the number of brick-and-mortar retail shops in city centres has decreased significantly, partly due to increased competition from e-commerce and partly due to declining demand. The decline of populations and population densities has reduced the number of potential retail customers, causing multiple local retailers to either close their businesses or move to more profitable locations. As a result, the local availability of products and services has declined in shrinking cities. The network of the consequences of e-commerce on the well-being of residents of shrinking communities is visualised in Fig. 6, and the most frequent concepts are discussed below.

Parallel to the decline in local availability of products and services, e-commerce has improved the remote availability of products and services in shrinking communities. E-commerce expands the selection of goods and thus improves consumption opportunities in shrinking cities, giving residents of shrinking and rural communities and regions access to diverse products similar to the access enjoyed by people in large cities. This equal access to products improves equality between different regions, but some delivery companies do not deliver packages to remote locations, causing spatial inequality.

The Covid-19 pandemic especially accelerated the development and expansion of online groceries. Ordering groceries online for delivery to homes or pick-up points saves time and makes everyday life easier. Online groceries can significantly improve the quality of life of elderly people in sparsely populated areas and enable them to live longer in their own homes.

Although e-commerce can be seen as contributing to the plight of local retail shops, it can also introduce new opportunities to local entrepreneurs. The environmental scanning revealed examples of local entrepreneurs successfully combining e-commerce with retail shops, enabling them to reach wider customer bases and improve their revenues.

## 4 Discussion

This study uncovered and assessed the impacts of digital transformation and ICT on residents' well-being in shrinking communities. Environmental scanning revealed the main themes of remote working, distance learning, distance services and e-commerce. Using environmental scanning and futures wheels, we identified multiple possible consequences of these themes on the well-being of shrinking communities’ residents.

Shrinking communities typically suffer from high rates of unemployment, mainly due to a lack of job opportunities, and unemployment has been connected to lower well-being in multiple previous studies (e.g. Ref. [18]). The rise of remote working improves employment opportunities in shrinking and rural communities, as the work in some occupations can be done entirely remotely. Remote working also enables multi-local living, which makes it possible for residents of large cities to spend longer periods in their second homes and summer cottages, typically in rural areas in shrinking municipalities. In the Finnish Free-Time Residence Barometer 2021, 43% of respondents stated that they had been working at their second home, and many of those respondents expressed a willingness to increase such work in the future [61]. Digitalisation has also enabled novel employment opportunities for youth who would otherwise have to move to larger cities in pursuit of employment.

Shrinking and rural communities typically have poor educational opportunities, and it is common for youngsters to move to major cities to pursue education, but distance learning offers new educational opportunities ranging from upper secondary school courses to complete university degrees. A higher educational level has previously been connected to higher levels of well-being [15], and improved educational possibilities can improve the well-being of shrinking communities’ residents. Higher education can also lead to higher income levels and better possibilities of remote working, which can contribute to improved overall well-being.

Remote services and e-commerce facilitate well-being and modern life in rural and shrinking communities, as comprehensive goods and services can be procured without travel to a nearby city. Improved remote services, such as remote healthcare and online groceries, are especially beneficial for elderly residents of shrinking communities, enabling them to live longer independently in their own homes. Multiple previous studies have shown that ICT increases the well-being of the elderly, for example, by supporting the mental and physical well-being of people over 80 [62] and enabling the elderly to achieve a sense of control and independence as well as other benefits that support their well-being [63]. The elderly may lack the necessary skills to use online services efficiently, potentially increasing inequality between younger and older residents, but Finland has support and educational schemes that aim to mitigate that problem. Overall, the expanding service and product selection enabled by digitalisation increases geographical equality between large growing cities and shrinking rural communities.

Finland's telecommunication networks are extensive, but this is not the case everywhere in the world. It has been estimated that nearly half the global population is unable to access the internet due to a lack of availability [64]. The lack of connectivity and/or coverage especially affects rural and low-income regions, even in developed countries [[64], [65], [66]], but many studies (e.g. Refs. [[64], [65], [66], [67]]) have attempted to identify solutions these issues and improve coverage and connectivity in rural and low-income areas.

Although the study found that ICT has many positive effects on people's well-being in shrinking rural regions, previous research has shown that it can also have negative or conflicting effects. For example, a study in Italy found that the expansion of distance learning during the Covid-19 pandemic increased computer anxiety and burnout among teachers [68]. Overall, studies have linked the use of ICT in organisations to stress, technostress, anxiety, strain and burnout, all of which pose challenges to well-being [[26], [27], [28],^69^,^70^]. However, some studies have yielded contradictory results [71]. For instance, the use of ICT can improve well-being, as it may enhance flexibility and efficiency as well as a sense of autonomy [72,73].

This study narrowed an existing research gap by advancing the understanding of digitalisation's impact on the well-being of shrinking communities. It represents a significant contribution to the research area of shrinking municipalities, offers a fresh perspective on an important topic and provides valuable insights into digitalisation's current and future consequences on well-being. Integrating the study of ICT and well-being in the context of shrinking rural municipalities and combining that with futures research methods represents a novel approach to studying these topics. To our knowledge, no other study has combined the three elements of shrinking rural communities, ICT and well-being to provide a comprehensive understanding of their future consequences on society. This study deliberately examined the effects of ICT in a very comprehensive manner to offer a broad overview of its impacts on well-being in the context of shrinking municipalities. The findings provide important information on the benefits and opportunities of digitalisation for residents of shrinking and rural communities, information that is especially useful for decision makers in those areas. The more local decision makers know about possible threats and opportunities for the future development of shrinking communities, the better the decisions they can take in the present.

Based on our results, it can be stated that digitalisation advances the United Nations Sustainable Development Goals (SDGs) [74] in the context of well-being in shrinking and rural communities. For instance, distance learning directly supports SDG 4, ‘Ensure inclusive and equitable quality education and promote lifelong learning opportunities for all’, by enabling location-independent education. Remote working supports SDG 8, ‘Decent work and economic growth’, and remote services contribute to SDG 3, ‘Good health and well-being’. Additionally, digitalisation and all the themes covered in this paper reduce inequalities between growing cities and shrinking rural areas, which directly connects to SDG 10, ‘Reduced inequalities within and among countries’. Previous studies have also identified the potential of ICT to create a more sustainable future. For instance, Wu et al. conducted a comprehensive literature review to identify correlations between ICT and the SDGs [75] and found that ICT has the potential to play significant and vital roles in supporting global economic, social and environmental sustainability [75]. In addition, the emergence of big data is believed have great potential in promoting sustainable development [67].

## 5 Limitations and future research

This study has several limitations that merit consideration. First, the results are strongly dependent on the data sources used, raising the question of whether newspapers were suitable for this study. Other data sources, such as blog posts, research articles or other newspapers, may have yielded different results. However, newspapers have been used as data sources in previous studies (e.g. Refs. [45,55,76]), justifying their use in this one.

The results may also be limited by the time period covered by the newspaper articles. Most of the local newspaper articles were published during the Covid-19 pandemic, which likely influenced the results. For instance, due to the pandemic, people in various industries transitioned to remote work on a large scale, and schools and educational institutions temporarily closed their doors and shifted to distance learning [77]. Visitor numbers in shopping centres and brick-and-mortar stores decreased as people sought to avoid contact, causing an increase in online shopping. These significant changes in society were extensively reported in newspapers during that time, which may have impacted the results of the study. Surprisingly, many of the identified future consequences were positive, although previous studies have shown that ICT also has negative effects on well-being [[26], [27], [28],^78^]. It may be that, during the pandemic, newspapers focused on new ways of operating and adapting to the situation, leading to a positive reporting bias.

Both the futures wheel and environmental scanning methods have limitations, as their effectiveness depends on the data quality [45,55,76] and the skills and experience of the individuals or teams employing these methods [55]. One limitation of the futures wheel method is that it can produce an extensive number of future consequences, making analysis and management challenging. Benckendorff has described other limitations of the futures wheel method, such as the speculative nature of the data, information overload and complex, time-consuming data analysis [46]. In this study, the limitations include the potential positive bias of the data and the possible bias of the expert team creating the wheels. Thus, the futures wheels presented in this article may fail to consider all the complex interdependencies between diverse phenomena, leading to oversimplifications and incomplete analysis.

Although this study focused on digitalisation's impacts on the well-being of residents of Finnish shrinking communities, the results may be partially generalisable to other Western countries experiencing similar trends connected to digitalisation. Finland has special characteristics, however, such as the widespread ownership of second homes and summer cottages, that make the associated results and impacts quite specific to Finnish shrinking municipalities. Large scale second home ownership in Finnish municipalities is covered, for instance, in Refs. [79,80] and the widespread trend of remote working from those locations [81–83]. That is, additional research on rural and shrinking communities in multiple countries is needed to fully assess the global impacts of digitalisation on rural well-being. It would also be interesting to conduct similar studies a few years after the end of the Covid-19 pandemic to fully assess the lasting effects of, for instance, the large-scale remote working period.

During the environmental scanning, a few additional emerging themes were identified that may impact well-being in shrinking communities in the long term, but those developments, such as autonomous mobility, are so far from realisation that they were not chosen for further analysis in this study. Autonomous mobility may, however, have a great impact on shrinking communities in the future, as it enables longer commuting and travel times and could thus bring new residents, seasonal residents, and tourists to rural areas. Digitalisation also enables other novel, currently available mobility services, such as scooter, bike, and electric vehicle (EV) sharing schemes, that are beneficial for both local residents and tourists [79]. EV sharing could also support the financial position of rural regions through utilization of EVs as local electricity storages and in different demand response schemes [79,80].

Future research should further study the impacts of such digitalisation technologies on the overall well-being of shrinking rural communities. Especially interesting would be to investigate the impact of different digitalisation technologies (from content creation to novel mobility services) on increased rural tourism and seasonal residency, as tourism and seasonal residents bring vitality to the whole region. Additionally, future research should consider both smaller rural and larger urban communities when examining the extensive impacts of digitalisation.

## 6 Conclusions

This study assessed the current and future consequences of digitalisation and ICT on the well-being of residents of shrinking communities in Finland. By employing futures research methods, including environmental scanning and futures wheels, we created a broad overview of the impacts of ICT on well-being in shrinking regions. According to our results, digitalisation and ICT have had a major impact on the well-being of residents of shrinking and rural communities in Finland. This impact has been mainly positive, and digitalisation can be seen as an important enabler of geographical equality. Digitalisation has made previously unavailable goods, services and employment and education opportunities available to residents of sparsely populated areas. In the future, digitalisation can further narrow the divide between large urban cities and shrinking rural communities, making it even easier for people to choose their living place based on personal preferences rather than employment and service availability.

## Author contribution statement

Annamari Kiviaho: Conceived and designed the experiments; Performed the experiments; Analysed and interpreted the data; Contributed reagents, materials, analysis tools or data; Wrote the paper. Johannes Einolander: Performed the experiments; Analysed and interpreted the data; Contributed reagents, materials, analysis tools or data; Wrote the paper.

## Data availability statement

Data will be made available on request.

## Additional information

No additional information is available for this paper.

## Declaration of competing interest

The authors declare that they have no known competing financial interests or personal relationships that could have appeared to influence the work reported in this paper.
